# Mucositis-associated bloodstream infections in adult haematology patients with fever during neutropenia: risk factors and the impact of mucositis severity

**DOI:** 10.1007/s00520-024-08776-w

**Published:** 2024-08-08

**Authors:** Nick A. de Jonge, Jeroen J. W. M. Janssen, Paula Ypma, Alexandra H. E. Herbers, Arne de Kreuk, Wies Vasmel, Jody M. W. van den Ouweland, Aart Beeker, Otto Visser, Sonja Zweegman, Nicole M. A. Blijlevens, Michiel A. van Agtmael, Jonne J. Sikkens

**Affiliations:** 1 Department of Haematology, Amsterdam University Medical Centers, Vrije Universiteit Amsterdam, De Boelelaan 1117, 1081 HV Amsterdam, the Netherlands; 2 Cancer Center Amsterdam, Treatment and Quality of Life, Amsterdam, the Netherlands; 3 Department of Haematology, Radboud University Medical Center, Geert Grooteplein Zuid 10, 6525 GA Nijmegen, the Netherlands; 4 Department of Haematology, HAGA Hospital, Els-Borst-Eilersplein 275, 2545 AA The Hague, the Netherlands; 5 Department of Haematology and Oncology, Jeroen Bosch Hospital, Henri Dunantstraat 1, 5223 GZ Hertogenbosch, Netherlands; 6 Department of Internal Medicine, OLVG, Jan Tooropstraat 164, 1061 AE Amsterdam, the Netherlands; 7 Department of Clinical Chemistry, Canisius-Wilhelmina Hospital, Weg Door Jonkerbos 100, 6532 SZ Nijmegen, The Netherlands; 8 Department of Internal Medicine, Spaarne Gasthuis, Spaarnepoort 1, 2134 TM Hoofddorp, the Netherlands; 9 Department of Medicine, Isala, Dokter Van Heesweg 2, 8025 AB Zwolle, the Netherlands; 10 Department of Internal Medicine, Amsterdam University Medical Centers, Vrije Universiteit Amsterdam, De Boelelaan 1117, 1081 HV Amsterdam, the Netherlands

**Keywords:** Fever, Neutropenia, Mucositis, Typhlitis, Bacteremia, Candidemia, Sepsis, Chemotherapy, Stem cell transplantation

## Abstract

**Purpose:**

Haematology patients with high-risk neutropenia are prone to mucosal-barrier injury-associated laboratory-confirmed bloodstream infections (MBI-LCBI). We assessed risk factors for MBI-LCBI including candidaemia in neutropenic haematology patients with fever.

**Methods:**

This prospective observational study was performed in six dedicated haematology units in the Netherlands. Eligible haematology patients had neutropenia < 500/mL for ≥ 7 days and had fever. MBI-LCBIs were classified according to Centers for Disease Control (CDC) definitions and were followed until the end of neutropenia > 500/mL or discharge.

**Results:**

We included 416 patients from December 2014 until August 2019. We observed 63 MBI-LCBIs. Neither clinical mucositis scores nor the blood level of citrulline at fever onset was associated with MBI-LCBI. In the multivariable analysis, MASCC-score (odds ratio [OR] 1.16, 95% confidence interval [CI] 1.05 to 1.29 per point decrease), intensive chemotherapy (OR 3·81, 95% CI 2.10 to 6.90) and *Pichia kudriavzevii* (formerly *Candida krusei*) colonisation (OR 5.40, 95% CI 1.75 to 16.7) were retained as risk factors for MBI-LCBI, while quinolone use seemed protective (OR 0.42, 95% CI 0.20 to 0.92). Citrulline level (OR 1.57, 95% CI 1.07 to 2.31 per µmol/L decrease), active chronic obstructive pulmonary disease (OR 15.4, 95% CI 1.61 to 14.7) and colonisation with fluconazole-resistant *Candida* (OR 8.54, 95% CI 1.51 to 48.4) were associated with candidaemia.

**Conclusion:**

In haematology patients with fever during neutropenia, hypocitrullinaemia at fever onset was associated with candidaemia, but not with bacterial MBI-LCBI. Patients with intensive chemotherapy with a low MASCC-score and colonisation with *Pichia kudriavzevii* had the highest risk of MBI-LCBI.

**Trial registration:**

ClinicalTrials.gov (NCT02149329) at 19-NOV-2014.

**Supplementary Information:**

The online version contains supplementary material available at 10.1007/s00520-024-08776-w.

## Introduction

Patients receiving myelotoxic treatment for haematological malignancies are at high risk for bloodstream infections (BSI) and sepsis, which brings about a significant risk of intensive care admission and treatment-related mortality [1]. Many neutropenic fever episodes coincide with an chemotherapy-induced inflammatory response of the gastro-intestinal mucosa, called mucositis [2]. This mucositis-associated gastro-intestinal inflammation results in mucosal barrier disruption which is thought to be the main cause of secondary bloodstream infections [3, 4]. On the other hand, no infectious cause is found in 30% to 60% of neutropenic fever episodes. It is hypothesized that these cases of fever of unknown origin are caused by mucositis-associated inflammation and not by infection, which questions the need for prolonged antibiotic therapy [5]. The uncertainty about the true cause of fever often results in continuation of antibiotic treatment and inherently overtreatment of a group of patients without infection [6, 7]. In order to improve appropriate use of antibiotics for fever during neutropenia, it is important to differentiate between infectious and non-infectious fever and be aware of the presence of risk factors for mucositis-associated bloodstream infections.[7] Previous retrospective studies reported that mucositis severity [3, 8–11], prolonged neutropenia, gastro-intestinal dysbiosis [12, 13], myeloablative conditioning before haematopoietic stem cell transplantation (HSCT) [9] and lack of antibiotic prophylaxis [14] are the most important risk factors for mucositis-associated bloodstream infections. However, there is a lack of prospective multicentre studies focusing on the first days after the onset of febrile neutropenia when the important decision whether or not to continue empirical antibiotics has to be made. In this study, we aimed to improve the understanding of fever during neutropenia by identifying risk factors for mucositis-associated bloodstream infections in haematology patients with high-risk fever during neutropenia.

## Methods

This prospective observational study was conducted between December 1st, 2014 and August 1st, 2019 in six hospitals in the Netherlands: one academic hospital (Amsterdam University Medical Centres [UMC], location Vrije Universiteit medical centre [VUmc]) and five regional teaching hospitals (OLVG, location West [Amsterdam], HAGA hospital [the Hague], Jeroen Bosch Ziekenhuis [‘s-Hertogenbosch], Spaarne Gasthuis [Hoofddorp] and Isala [Zwolle]). All centres had dedicated haematology units. This study was performed in line with the principles of the Declaration of Helsinki and was approved by the research ethics committee of the Amsterdam UMC, location VUmc on October 29th 2014 and the ethical bodies of each of the participating centres. All participants gave written informed consent.

### Participants

Eligible patients were adults (≥ 18 years) treated for a haematological malignancy with intensive chemotherapy or hematopoietic stem cell transplant (HSCT) and had fever, (defined as one tympanic temperature measurement of ≥ 38·5 °C or two measurements of ≥ 38·0 °C measured at least 2 h apart) and high-risk neutropenia (< 500 cells/mL expected to last for ≥ 7 days) at onset of fever. All patients with expected high-risk neutropenia were asked informed consent at admission (i.e. before fever occurred). Only patients that experienced fever during neutropenia were analysed. (appendix p.4). Patients with fever of unknown origin after 72 h of empirical antibiotic treatment participated in a randomised clinical trial investigating short or extended treatment of a carbapenem in haematology patients with febrile neutropenia of unknown origin (NCT02149329) [15]. Those patients who were ineligible for participation in the main trial due to infection or septic shock before day 3 after fever onset, were still eligible for this study.

### Procedures

At fever onset (day 0; T0), two sets of blood cultures were drawn from the central venous catheter or by venepuncture. Follow-up blood cultures were taken daily as long as fever persisted, or up to 7 days after fever onset. Laboratory assessment included a complete blood count, renal and liver function tests and were performed at least three times a week. Presence and severity of oropharyngeal and intestinal mucositis were scored by trained nurses on day 1 or 2 after fever onset. Citrulline, an amino acid produced by enterocytes, is a biomarker of intestinal integrity [16]. Very low values, particularly < 10 mmol/L, are frequently observed in patients with (e.g. chemotherapy-induced) mucosal injury and can therefore be regarded as a measure for quantifying intestinal mucositis [11, 17]. Blood samples for citrulline were drawn the first morning after fever onset (T1) in four of six centres and measured as previously described [18].

### Definitions

Bacteraemia was classified as laboratory confirmed bloodstream infection (LCBI) using the Centers for Disease Control (CDC) National Healthcare Safety Network (NHSN) criteria. (appendix, p.2) [19] According to these CDC-criteria, the mucosal barrier injury laboratory–confirmed bloodstream infection (MBI-LCBI) was defined as the isolation from blood of an intestinal organism from the NHSN MBI organism list in the presence of neutropenia, which was an prerequisite for participation in this study. Bacteraemia was classified as LCBI1 if a recognised pathogen was isolated at least once; or LCBI2 if a common commensal pathogen (e.g. coagulase-negative staphylococci) was isolated on two separate occasions.

Oral mucositis was graded according to the Oral Mucositis Grading Score of the World Health Organization [20]. Intestinal mucositis grading was performed based on a modified scoring of diarrhoea according to the Common Terminology Criteria for Adverse Events (CTCAE) version 5·0 with adjustment for stool consistency and severity of abdominal pain or cramps. (appendix, p.3) [21].

### Antibiotic prophylaxis and treatment

All participants received antibiotic and antifungal prophylaxis (appendix, p.3). Empirical antibiotic treatment for fever during neutropenia consisted of either imipenem-cilastatin 500 mg QID or meropenem 1000 mg TID. Treatment length in patients with fever of unknown origin after day 3 was allocated by randomisation as described previously.[15] Antibiotic treatment duration was according to the discretion of the treating physician for patients not included in the randomised trial.

### Statistical analysis

Continuous variables were reported as means with 95% confidence intervals or medians with interquartile ranges as appropriate. Logistic regression was used to investigate association between MBI-LCBI and clinically relevant covariates. Relevant covariates were those previously published in literature and likely to impact mucositis severity or risk of secondary bloodstream infection. These included age, sex, haematological diagnosis and treatment, severity of illness, fever status at day 3, mucositis severity, use of antimicrobial prophylaxis and positive colonisation cultures. All included covariates are summarised in the appendix (p.7 to 10).

All covariates with *p* < 0.2 were eligible for inclusion in the multivariable model which was subsequently determined using backward selection with a *p*-value < 0.05 threshold. We compared MBI-LCBI incidence over time using generalized estimating equations with a Poisson distribution.

## Results

### Participants and baseline characteristics

We screened 798 patients admitted to the hospital with expected high-risk neutropenia, of which a total of 416 patients experienced documented fever between December 1st 2014 and July 1st 2019 (appendix, p.4). Median age was 59 years (interquartile range [IQR] 52─65) and 251 (62%) were male. Haematopoietic stem cell transplantation (HSCT) was performed in 271 (65%) patients. Fever occurred at a mean of 11.6 days (95% confidence interval [CI] 11·0 to 12.2) after onset of chemotherapy, and the median duration was 3.7 days (IQR 3.5 to 40). Multinational Association for Supportive Care in Cancer (MASCC) score was 20 (IQR 18–21, possible values 0 to 26, with lower values indicating higher risk for poor outcome) at the onset of fever, indicating that the population was at high-risk of complications (Table 1).

**Table 1:** Patient characteristics during fever onset

	All (*N* = 416)
Age (year)—median (IQR*)	59 (52─65)
Sex assigned at birth—no. (%)	
male	251 (60.3%)
female	165 (39.7%)
Haematological condition—no. (%)	
multiple myeloma	149 (35.8%)
acute myeloid leukaemia	126 (30.3%)
lymphoma	84 (20.2%)
myelodysplastic syndrome	37 (8.8%)
other◊	20 (4.8%)
Chemotherapy	145 (34.8%)
HSCT^#^—no. (%)	271 (65.1%)
autologous	216 (51.9%)
allogeneic	55 (13.2%)
Reduced intensity conditioning	39 (9.4%)
Myeloablative conditioning	16 (3·8%)
ECOG Performance at onset of fever†—median (IQR)	2 (2─3)
MASCC-score‡—median (IQR)	20 (18─21)
Oral mucositis WHO-score—median (IQR)^∞^	
grade 0	228 (54.8%)
grades 1–2	138 (33.2%)
grades 3–4	48 (11.5%)
Intestinal mucositis grade—CTCAE-score ^Θ^	
grade 0	133 (32.0%)
grades 1–2	243 (58.4%)
grade 3	27 (6.5%)
White blood cell count (× 10e9/L)	0.10 (0.00─0.30)
Neutrophil count (× 10e9/L)	0.00 (0.00─0.01)
Neutropenia duration (days, median (IQR)	
before fever onset	3 (1─7)
after fever onset	10 (6─18)

*Interquartile range

^◊^Others include acute lymphoblastic leukaemia (*n* = 10), myelofibrosis (*n* = 5), chronic myeloid leukaemia (*n* = 3) and chronic lymphocytic leukaemia (*n* = 1) blastoid plasmacytoid dendritic cell neoplasm (*n* = 1)

^#^Haematopoietic stem cell transplantation

^†^Eastern Cooperative Oncology Group

^‡^Multinational Association of Supportive Care in Cancer

^∞^Two cases had missing data

^Θ^13 cases had missing data

### Bloodstream infections

A total of 129 bloodstream infections (BSIs) were documented in 105 of 416 patients (25.2%). In 88 (68.8%) BSIs, blood cultures were positive within 24 h and in 109 (84.5%) within 72 h after start of fever. None of the isolated pathogens after 72 h from the start of fever was susceptible to the empirical antibiotic regimen (appendix, p.5 to 6). Sixty-three cases of BSI were classified as mucositis-associated bloodstream infections (MBI-LCBI) in 60 patients. Central line–associated bloodstream infections were caused by 21 BSIs with a recognized pathogen (LCBI1) and 45 BSIs with a common commensal pathogen (LCBI2). The most frequently identified pathogens in the MBI-LCBIs were *Enterococcus faecium* (*n* = 31; 49.2%), viridans group streptococci (*n* = 8; 12.7%) and *Escherichia coli* (*n* = 6; 9.5%). (appendix, p.5) Of the 120 bacteria isolated in blood, 11 (9.2%) of these were preceded by a colonisation culture with the same pathogen.

Candidaemia occurred in nine patients (2.1%), with isolation of *Pichia kudriavzevii* (formerly known as *Candida krusei*) (*n* = 3), *Candida tropicalis* (*n* = 3), *Candida albicans* (*n* = 1), *Candida inconspicua* (*n* = 1) and *Candida norvegensis* (*n* = 1) as causative pathogens. (appendix, p.5) In six of nine cases (67%) of candidaemia, the *Candida* spp. isolated in blood was previously cultured in surveillance cultures.

### Risk factors for bloodstream infections

Results of the univariable analysis are shown in the appendix p.7 to 10. In the multivariable analysis, intensive chemotherapy vs. HSCT (OR 3.81, 95% CI 2.10 to 6.90), MASCC-score (odds ratio [OR] of 1.16, 95% CI 1.05 to 1.28 per point decrease), and colonisation with *Pichia kudriavzevii* (formerly *Candida krusei,* OR 5.40, 95% CI 1.75 to 16.7) were significantly associated with MBI-LCBI. Conversely, quinolone-based prophylaxis protected against MBI-LCBI (OR 0.42, 95% CI 0.20 to 0.92) (Table 2).

**Table 2:** Multivariable regression model of MBI-LCBI

	OR (95% confidence interval)
Chemotherapy vs. HSCT	3.81 (2.10 to 6.90)
*Pichia kudriavzevii* colonisation	5.40 (1.75 to 16.7)
MASCC score (per point decrease)	1.16 (1.05 to 1.28)
Quinolone prophylaxis use	0.42 (0.20 to 0.92)

*MBI-LCBI* mucosal barrier injury–associated laboratory confirmed bloodstream infection, *HSCT* haematopoietic stem cell transplantation, *MASCC score* Multinational Association for Supportive Care in Cancer, *OR* odds ratio.

We separately analysed patients that experienced bacterial MBI-LCBIs (52 patients) and those caused by *Candida spp*. (nine patients). One patient experienced both. Multivariable analysis revealed that only MASCC-score (OR 1.17, 95% CI 1.30 to 1.06 per point decrease) and intensive chemotherapy (vs. HSCT) (OR 3.21, 95% CI 1.75 to 5.87) remained significantly associated with bacterial MBI-LCBI. (appendix, p.10).

The following three variables were retained in the final candidaemia multivariable regression model: citrulline level (OR 1.57, 95% CI 1.07 to 2.31 per µmol/L decrease), active COPD (OR 15.4, 95% CI 1.61 to 14.7) and colonisation with fluconazole-resistant *Candida spp*. (OR 8.54, 95% CI 1.51 to 48.4) (Table 3).

**Table 3:** Multivariable regression model of candidaemia

	OR (95% confidence interval)
Active COPD	15.4 (1.61–14.7)
Fluconazole-resistant *Candida* spp*.* colonisation	8.54 (1.51–48.4)
Citrulline level (per µmol/L decrease)	1.57 (1·07–2.31)

*COPD* chronic obstructive pulmonary disease, *OR* odds ratio.

In a post hoc analysis, we excluded BSIs with *Pichia kudriavzevii* as MBI-LCBI to investigate if colonisation with this pathogen was also associated with other mucositis-associated BSIs. However, *Pichia kudriavzevii* colonisation was no longer associated with MBI-LCBI after excluding BSIs with this pathogen (OR 1.83, 95% CI 0.49 to 7.12), while the other previously associated factors remained significant (appendix, p.10).

### Timing of MBI-LCBIs

After adjusting for MASCC score, intensive chemotherapy, prophylactic quinolone use and colonisation with *Pichia kudriavzevii*, the incidence rate ratios (IRR) for risk of MBI-LCBI were significantly increased in the second (6.05 [2.20 to 16.7]) and third (10.5 [3.66 to 30.1]) week after start of chemotherapy (*p* < 0·001) compared to the first week (Table 4). Poisson regression assumes independence of outcomes, which is probably not met in our cohort, as the risk of a subsequent MBI-LCBI event in the same time period may be either higher (e.g. due to mucositis severity) or lower (e.g. due to antibiotic therapy) after the first event. Although this was rare in our cohort (only one instance), we used a GEE with a binomial distribution as sensitivity analysis to assess the impact on our results. In this analysis, the case with multiple events in the same time period was seen as one event. This analysis showed comparable odd ratios (appendix p. 11).

**Table 4:** Incidence of MBI-LCBI

	Period from start of chemotherapy				
	Days 1–7	Days 8–14	Days 15–21	Days 22–28	Day > 28
No. of patients	416	411	393	204	97
No. of MBI-LCBI	4	24	29	3	3
Time at risk in days^§^	2893	2870	1871	859	910
Incidence rate per 1000 days at risk (95% CI)	1.38 (0.44–3.34)	8.36 (5.48–12.3)	15.5 (10.6–22.0)	3.49 (0.89–9.51)	3.30 (0.84–8.97)
IRR crude (95% CI)	1 (ref.)	6.05 (2.18–16.8)	11.2 (3.93–32.0)	2.53 (0.57–11.2)	2.39 (0.56–10.1)
IRR adjusted^*^ (95% CI)	1 (ref.)	6.05 (2.20–16.7)	10.5 (3.66–30.1)	2.05 (0.46–9.23)	1.83 (0.41–8.17)

^§^Time at risk from start of chemotherapy to end of neutropenia or discharge

*Adjusted for MASCC score, chemotherapy, prophylactic quinolone use and colonisation with *Pichia kudriavzevii*

*IRR* incidence rate ratio, *MBI-LCBI* mucosal barrier injury–associated laboratory confirmed bloodstream infection

### Colonisation cultures and resistance

Streptococci colonisation was present in 63.7% (265/416) of patients, of whom 5.7% (15/265) had colonisation penicillin-resistant streptococci. Of the 60 patients with enterococci colonisation, two (3.2%) of these were vancomycin-resistant enterococci (VRE). *Staphylococcus aureus* was cultured in 8.7% (36/416) of patients.

Of the Gram-negative microorganisms, *Escherichia coli* and *Klebsiella* spp. were cultured in surveillance cultures in 52.2% (217/416) and 19.2% (80/416) of patients respectively. Of all 416 patients, 36 (8.7%) were active or previous carriers of a multiresistant Gram-negative pathogen, including extended spectrum beta-lactamase-producers.

*Candida* spp. colonisation was documented in 56.5% (235/416) of patients and 27.9% of all *Candida* cultures were resistant to fluconazole and none to amphotericin-B and triazoles. The risk of candidaemia was 5.2% (5 of 96) patients in patients with previous fluconazole-resistant *Candida* colonisation versus 1.3% (4/320) with no fluconazole resistant colonisation (OR 4.34, 95% CI 1.14–16.5 in the univariable analysis).

### Mucositis

Clinical oropharyngeal mucositis was present during the first 3 days after fever onset in 186 of 416 (44.8%), of which 48 (25.8%) was deemed severe (grades 3–4). Signs of intestinal mucositis were present in 270 (64.5%) patients of which 27 (10.0%) were severe cases.

Citrulline levels were available for 248 patients and were collected at a median of 12 days (IQR 10 to 16) after the start of chemotherapy. The median citrulline level on day 1 after fever onset was 8.2 (IQR 5.2 to 12.3) µmol/L. Citrulline levels were below < 10 µmol/L (indicative of severe mucosal integrity disruption) in 62.9% (156/248) of patients. Using citrulline level of < 10 µmol/L as the cut-off level for mucositis, the sensitivity and specificity of clinical mucositis (the highest grade of clinically assessed oropharyngeal or intestinal mucositis ≥ grade 1) were 83.3% and 39.1%, respectively with corresponding negative and positive predictive values of 69.9% and 58.0%. ROC analysis revealed an area under the curve (AUC) of 0.612 (95% CI 0.538 to 0.687). A weak correlation was found between mucositis scores and citrulline level as continuous variable (rho =  − 0·342 [95% CI − 0.227 to 0.448, *p* < 0·001) indicating that clinical mucositis scores were a poor indicator of mucosal damage.

## Discussion

In this prospective observational study, we identified intensive chemotherapy, low MASCC score and *Pichia kudriavzevii* (formerly *Candida kruseï*) colonisation as risk factors for mucosal barrier injury associated laboratory confirmed bloodstream infection (MBI-LCBI) in haematology patients with fever during neutropenia. MBI-LCBIs particularly occurred in the second and third week after chemotherapy. This period coincides with the typical period for chemotherapy-induced mucositis, indicating that severe disruption of gut mucosal integrity leads to secondary bacteraemia of gut microbiota [4, 9, 12, 17]. In our study, neither mucositis severity at fever onset, nor neutropenia duration was an independent risk factor for MBI-LCBI, which is in contrast with previous studies [8–11]. There might be a few reasons for this discrepancy. First, mucositis was measured at fever onset, so fever in blood-culture negative cases may alternatively have been caused by mucositis-associated inflammation [5]. This is supported by a previous big-data analysis study that showed that mucositis was associated with fever, but not with BSI [22]. Our data also supports the hypothesis that the majority of fevers is caused by mucositis as 62·9% of patients had a severely decreased citrulline level, but only 15% of patients had mucositis-associated bloodstream infections. This contradictory finding may be explained by the fact that 98% of patients in our study received quinolone based antibiotic prophylaxis, which may have protected against MBI-LCBI occurrence. This finding is consistent with a Cochrane meta-analysis of a total of 935 patients concluding that quinolone-based prophylaxis reduced the number of Gram-negative bacteraemias in comparison to cotrimoxazole [14].

We found that decreasing MASCC-score, analysed as continuous variable, was associated with MBI-LCBI. However dichotomous MASCC-score, using the commonly used cut-off ≥ 21 for high risk and < 21 for low risk of complications, was not. This is probably due to the loss of power associated with dichotomizing continuous variables. Likewise, two previous studies showed that a MASCC-score < 21 had a low sensitivity of 42% to 46% for bacteraemia [23, 24]. One study reported that the addition of bacteraemia status to the MASCC-score did not meaningfully improve prediction of complications [23].

Our finding that 84.5% of bloodstream infections were identified in the first 3 days after fever onset and that none of the bacteraemias after day 3 were susceptible to empirical carbapenems support early discontinuation of empirical antibiotic treatment in culture-negative fever episodes. This adds context to the existing evidence on the clinical safety of early de-escalation of broad-spectrum antibiotics shown in two randomized controlled trials (of which one is part of the same project as the current study) [15].

Identification of patients with severe mucositis can be difficult, as shown by the low agreement between the clinical assessment of oral and intestinal mucositis and citrulline levels. In particular, the low specificity of the clinical scores in our study likely produced a high false positive rate [25]. However, the clinical relevance of grading mucositis severity to estimate the risk of mucositis-associated bacteraemia in these patients may be less than initially thought, as both the clinical mucositis examination and citrulline levels where not associated with MBI-LCBI risk and thus poorly reflect the true risk of secondary bloodstream infections from the gut.

A subgroup of MBI-LCBI is those with candidaemia. These cases particularly occurred in the setting of severe mucositis (reflected by hypocitrullinaemia), in patients with fluconazole-resistant *Candida* spp. in surveillance cultures. Patients with active chronic obstructive pulmonary disease had an increased risk of candidaemia, which might be caused by the impact of tobacco use or corticosteroid inhalation, which were both not measured in this study. While *Candida* spp. colonisation was observed in 56% of patients, the majority of candidaemia cases (six of nine cases) were preceded by colonisation with the same *Candida* spp*.*, highlighting the informative value of surveillance cultures and suggesting a role for enteral or systemic antifungal therapy in the case of previous fluconazole-resistant *Candida* spp*.* colonisation.

The strengths of the current study are the prospective nature and the multicentre design including data from both academic and teaching hospitals in the Netherlands. The interpretation and generalisability of the results may be influenced by some limitations. First, all patients received antimicrobial prophylaxis, which is not standard practice in all countries, despite its proven effectiveness [14, 26]. Second, antibiotic prophylaxis strategies varied within the study population possibly influencing the results, although this does reflect the *real-world* situation.

In conclusion, we found that mucositis-associated bloodstream infections in adult haematology patients with fever during neutropenia are more common in those with lower MASCC scores and previous colonisation with *Pichia kudriavzevii,* and particularly occurred in the second and third week after starting intensive chemotherapy. Patients colonised with *Candida* spp. resistant to prophylactic fluconazole and those with active COPD were at increased risk of candidaemia. Citrullin measurement seems to have no place in the work up febrile neutropenia to estimate the risk mucositis-associated *bacteraemia.* However, low citrulline levels and *Candida* colonisation in surveillance cultures were associated to candidaemia. The value of targeted antifungal prophylaxis in patients with a low citrulline level at fever onset combined with a positive *Candida* surveillance culture will have to be explored.

## Supplementary Information

Below is the link to the electronic supplementary material.

Supplementary file1 (PDF 501 KB)

## Data Availability

No datasets were generated or analysed during the current study.
