# Supplementary material for: Mucositis-associated bloodstream infections in adult haematology patients with fever during neutropenia: risk factors and the impact of mucositis severity
Source: Support Care Cancer. 2024 Aug 8;32(9):579. doi: 10.1007/s00520-024-08776-w (PMC11310222; doi:10.1007/s00520-024-08776-w)
Supplement: Supplementary file 1 — Supplementary file1 (PDF 501 KB) [file 520_2024_8776_MOESM1_ESM.pdf]

Supplemental appendix

Contents

Definitions 2

Supplemental Table 1 ─ Common Terminology Classification for Adverse Events with adjustment for mucositis 3

Supplemental Table 2 ─ Standard antimicrobial prophylaxis 3

Supplemental Figure 1 ─ Patient screening and inclusion 4

Supplemental Table 3 ─ Pathogens cultured in blood per BSI type 5

Supplemental Figure 2 ─ Bloodstream infection occurrence after start of the first fever episode 5

Supplemental Table 4 ─ Univariable and multivariable regression model of MBI-LCBI 7

Supplemental Table 5 ─ Univariable and multivariable regression model of candidaemia 9

Supplemental Table 6 ─ Multivariable regression model of bacterial MBI-LCBI pathogens 10

Supplemental Table 7 ─ Multivariable regression model of candidaemia after exclusion of *Pichia kudriavzevii* BSIs 10

Supplemental Table 8 – Sensitivity analysis of the incidence of MBI-LCBI after start of chemotherapy: comparison of binomial vs. Poisson generalised estimating equations 11

Definitions

**Centers for Disease Control (CDC) National Healthcare Safety Network (NHSN) criteria for classification of bacteraemias**	
LCBI 1	1. Patient of any age has a recognized bacterial or fungal pathogen, not included on the NHSN common commensal list.<br><br>**AND**<br><br>2. Organism(s) identified in blood is not related to an infection at another site.
LCBI 2	1. Patient of any age has at least one of the following signs or symptoms: fever (>38.0oC), chills, or hypotension.<br><br>**AND**<br><br>2. Organism(s) identified in blood is not related to an infection at another site.<br><br>**AND**<br><br>3. The same NHSN common commensal is identified by a culture from two or more blood specimens collected on separate occasions
MBI-LCBI	1. Patient of any age fully meets LCBI 1 criterion<br><br>**AND**<br><br>2.<br>A. With at least **one** blood specimen with **ONLY** intestinal organisms from the NHSN MBI organism list identified by culture or non-culture based microbiologic testing method<br><br>**OR**<br><br>B. With at least two matching blood specimens with ONLY Viridans Group Streptococcus and/or Rothia spp. alone but no other organisms identified by culture.<br><br>**AND**<br><br>3. Meets one of the following:<br>A. Is an allogenic HSCT recipient within the past year with either grade III or IV gastrointestinal graft versus host disease during the same hospitalisation, or has ≥1-liter diarrhea in a 24-hour period (or ≥20 mL/kg in a 24-hour period for patients<br><br>**OR**<br><br>*B.* Is neutropenic, defined as at least two separate days with ANC† and/or WBC values Isolation from blood of an intestinal organism from the NHSN MBI organism list in the presence of neutropenia, , which was an inclusion criterion for the trial
ANC – absolute neutrophil count; NHSN – National Healthcare Safety Network; *LCBI – laboratory-confirmed bloodstream infection; MBI – musical barrier injury; WBC – white blood cell count*	

- Polymicrobial bloodstream infections belonging to the same LCBI group according to CDC-NHSN criteria were classified as a single event when isolated on the same day.

Supplemental Table 1 ─ Common Terminology Classification for Adverse Events with adjustment for mucositis

Grade	CTCAE term diarrhoea	Addition for mucositis
**1**	Increase of <4 stools per day over baseline; Mild increase in ostomy output to baseline	Neither watery stool nor cramps or abdominal pain
**2**	Increase 4 – 6 stools per day over baseline; moderate increase in ostomy output compared to baseline; limiting instrumental ADL	Either watery stool consistency or cramps or abdominal pain
**3**	Increase of ≥7 stools per day over baseline; hospitalization indicated; severe increase in ostomy output compared to baseline; limiting self-care ADL	Watery stool consistency with cramps or abdominal pain requiring pain relief
**4**	Life-threatening consequences; urgent intervention indicated	

Supplemental Table 2 ─ Standard antimicrobial prophylaxis

Centre	1	2	3	4	5	6
Ciprofloxacin	X	X		X	X	
Norfloxacin			X			X
Tobramycin (oral)	X	X		X	X	
Pheneticillin	X	X	X	X		X
Fluconazole	X	X		X	X	
Itraconazole			X			X
Amphotericin-B (oral)						X

Antibiotic prophylaxis regimen was switched in case of resistant microorganisms in weekly taken pharyngeal and rectal surveillance cultures

Supplemental Figure 1 ─ Patient screening and inclusion

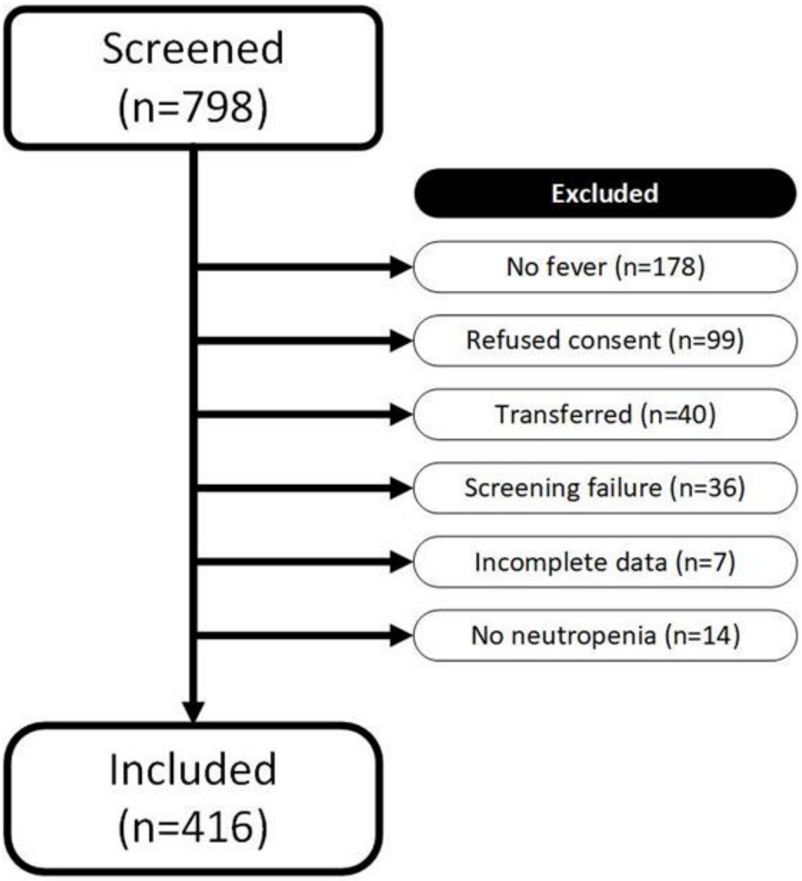

Supplemental Table 3 ─ Pathogens cultured in blood per BSI type

	All	≤1 days after previous fever onset	>3 days after previous fever onset
	No. of BSIs	No. of BSIs	No. of BSIs
MBI-LCBI			
Enterococcus faecium	31	16	7
Streptococcus viridans group	8	8	
Escherichia coli	6	6	
Pichia kudriavzevii	3	2	1
Candida tropicalis	3	1	1
Klebsiella pneumoniae	3	2	
Serratia marcescens	3	3	
Enterococcus faecalis	2	1	
Other◊	5	4	
LCBI1			
Stenotrophomonas maltophilia	9	4	2
Acinetobacter spp.	4	2	
Pseudomonas aeruginosa	2	2	
Pseudomonas spp. (non-aeruginosa)	2	1	
Moraxella osloensis	2	2	
Other ∫	4	4	
LCBI2			
Staphylococcus epidermidis	28	22	3
Staphylococcus haemolyticus	15	8	6
Staphylococcus hominis	4	2	1
Other§	3	3	

◊ Other MBI-LCBI organisms: *Candida albicans, Candida inconspicua, Candida norvegensis, Enterococcus casseliflavus* and *Proteus mirabilis*
∫ Other LCBI1 organisms: *Chryseobacterium indologenes, Roseomonas mucosa, Brevibacterium casei*
§ Other LCBI2 organisms: *Bacillus spp., Corynebacterium spp. Micrococcus spp.*

Abbreviations: BSI bloodstream infection; MBI-LCBI – mucosal barrier injury-associated laboratory confirmed bloodstream infection; LCBI1 laboratory confirmed bloodstream infection with recognized pathogen; LCBI2 laboratory confirmed bloodstream infection with common commensal

Supplemental Figure 2 ─ Bloodstream infection occurrence after start of the first fever episode

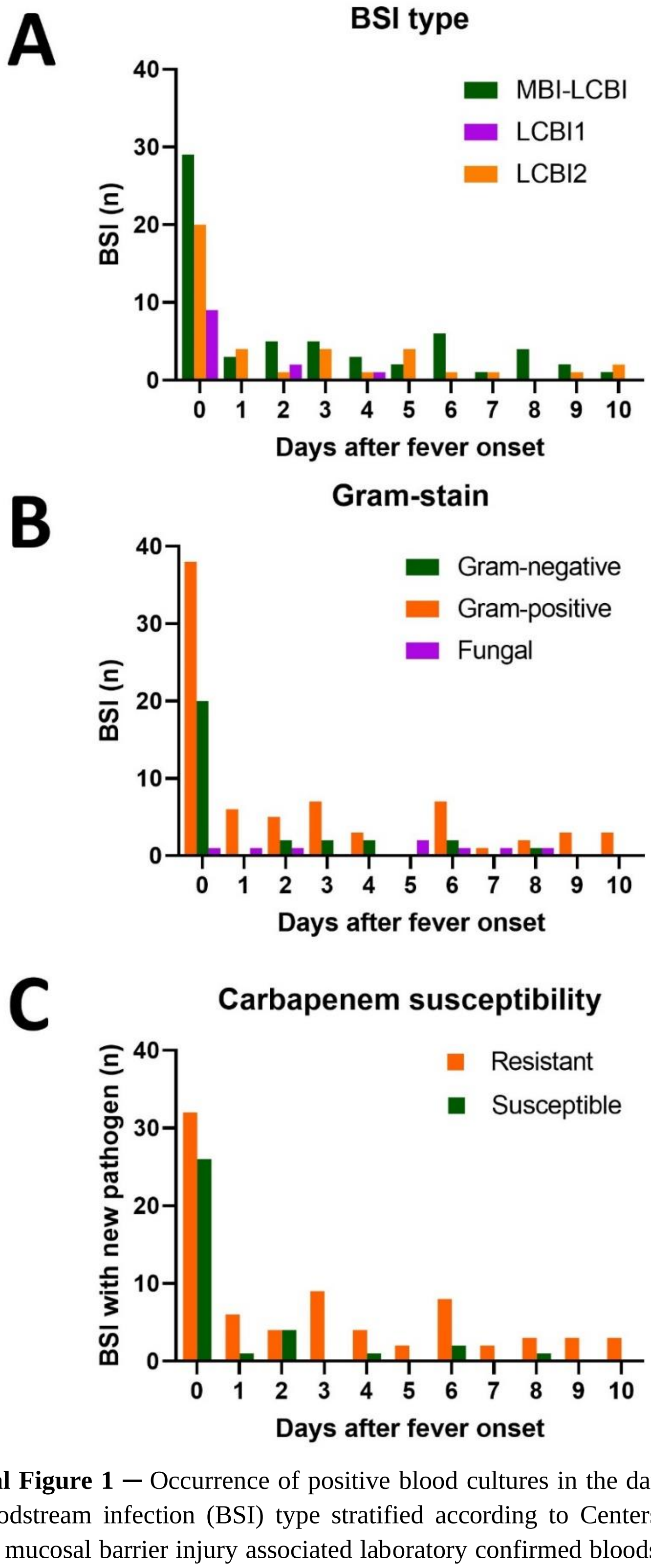

Supplemental Figure 1 ─ Occurrence of positive blood cultures in the days after start of fever (day 0). Panel A: bloodstream infection (BSI) type stratified according to Centers for Disease Control (CDC) definitions of mucosal barrier injury associated laboratory confirmed bloodstream infection (MBI-LCBI), BSI with a recognized pathogen (LCBI1) or common commensal pathogen (LCBI2). Panel B: BSIs stratified per Gram-stain of identified pathogen. Panel C: BSIs stratified according to susceptibility of the empirical antibiotic therapy with carbapenems.

Supplemental Table 4 ─ Univariable and multivariable regression model of MBI-LCBI

Factor	MBI-LCBI		OR	95% CI
**Univariate model**	**Group with factor (n/N.%)**	**Group without factor (n/N.%)**		
Age (years, [mean. 95% CI]	58.5 (55.2 – 61.8)	56.2 (54.9 - 57.5)	1.02	0.99 – 1.04
Male vs. female	40 / 258 (15.5%)	20 / 158 (12.7%)	1.27	0.71 – 2.27
Haematological diagnosis				
AML	28 / 126 (22.2%)	32 / 290 (11.0%)	2.30	1.32 – 4.03
MM	9 / 149 (6.0%)	51 / 267 (19.1%)	0.27	0.13 – 0.57
Lymphoma	9 / 84 (10.7%)	51 / 332 (15.4%)	0.66	0.31 – 1.40
MDS	10 / 37 (27.0%)	50 / 379 (13.2%)	2.44	1.11 – 5.34
Other	4 / 20 (20.0%)	56 / 396 (14.1%)	1.52	0.49 – 4.71
Clinic				
Hospital 2 vs. 1	3 / 79 (3.8%)	37 / 181 (20.4%)	0.15	0.05 – 0.52
Hospital 3 vs. 1	3 / 43 (7.0%)		0.29	0.09 – 1.00
Hospital 4 vs. 1	8 / 35 (22.9%)		1.15	0.48 – 2.75
Hospital 5 vs. 1	2 / 18 (11.1%)		0.49	0.11 – 2.21
Hospital 6 vs. 1	7 / 60 (11.7%)		0.51	0.22 – 1.22
Intensive chemotherapy vs. HSCT	36 / 144 (25.0%)	24 / 272 (8.8%)	3.44	1.96 – 6.05
Autologous vs. allogeneic HSCT	16 / 216 (7.4%)	8 / 56 (14.3%)	0.48	0.19 – 1.19
Thrombocyte count (x10^9/mL)	13.5 (10.2 – 17.9)	17.8 (16.1 – 19.8)	0.76	0.58 – 1.00
ECOG score[¥]				
1 vs. 0	12 / 72 (16.7%)	5 / 28 (17.9%)	0.92	0.29 – 2.90
2 vs. 0	19 / 134 (14.2%)		0.76	0.26 – 2.24
3 vs. 0	21 / 171 (12.3%)		0.64	0.22 – 1.88
4 vs. 0	3 / 9 (33.3%)		2.30	0.42 – 12.5
MASCC (per point decrease [mean. 95% CI])	18.8 (17.9 – 19.7)	19.9 (19.6 – 20.1)	1.14	1.04 – 1.26
MASCC <21 vs. ≥21 points	49 / 322 (15.2%)	11 / 94 (11.7%)	1.35	0.67 – 2.72
Burden of illness				
Moderate vs. mild/no symptoms	21 / 116 (18.1%)	36 / 290 (12.4%)	1.56	0.87 – 2.81
Severe vs. mild/no symptoms	3 / 10 (30.0%)		3.02	0.75 – 12.2
Hypotension	2 / 8 (25%)	58 / 408 (14.2%)	2.01	0.40 – 10.2
Active COPD	3 / 8 (37.5%)	57 / 408 (14.0%)	3.70	0.86 – 15.9
Previous fungal infection	5 / 24 (20.8%)	55 / 392 (14.0%)	1.61	0.58 – 4.50
Dehydration	29 / 196 (14.8%)	31 / 220 (14.1%)	1.06	0.61 – 1.83
Inpatient vs. outpatient status	57 / 391 (14.6%)	3 / 25 (12.0%)	1.25	0.36 – 4.32
Age ≥ 60 years	37 / 203 (18.2%)	23 / 213 (10.8%)	1.84	1.05 – 3.23
Fever after day 3	34 / 217 (15.7%)	26 / 199 (13.1%)	1.24	0.71 – 2.15
Oral Mucositis[◊]				
Grade 1-2 vs. 0	10 / 138 (7.2%)	44 / 228 (19.3%)	0.33	0.16 – 0.67
Grade 3-4 vs. 0	5 / 48 (10.4%)		0.49	0.18 – 1.30
Intestinal mucositis[∫]				
Grade 1-2 vs. 0	32 / 243 (13.2%)	19 / 133 (14.3%)	0.91	0.49 – 1.68
Grade 3-4 vs. 0	7 / 27 (25.9%)		2.10	0.78 – 5.64
Citrulline [ϴ] (per □mol/L decrease [mean. 95% CI])	8.59 (6.71 – 10.47)	9.40 (8.65 – 10.15)	1.03	0.97 – 1.09
Citrulline < 10 □mol/L [ϴ]	12 / 92 (13.0%)	37 / 156 (23.7%)	2.07	1.02 – 4.22
Prophylaxis				
Quinolone	48 / 371 (12.9%)	12 / 45 (26.7%)	0.41	0.20 – 0.85
TMP/SMX (960 mg BID)	2 / 18 (11.1%)	58 / 398 (14.6%)	0.73	0.16 – 3.27
Colistin	7 / 39 (17.9%)	53 / 377 (14.1%)	1.34	0.56 – 3.19

Tobramycin*	43 / 301 (14.3%)	17 / 115 (14.8%)	0.96	0.52 – 1.76
Amphotericin-B*	13 / 84 (15.5%)	47 / 332 (14.2%)	1.11	0.57 – 2.16
Fluconazole	37 / 266 (13.9%)	23 / 150 (15.3%)	0.89	0.51 – 1.57
Triazole¶	11 / 79 (13.9%)	49 / 337 (14.5%)	0.95	0.47 – 1.93
Colonization cultures				
Escherichia coli	31 / 217 (14.3%)	29 / 199 (14.6%)	0.98	0.57 – 1.69
Klebsiella pneumoniae	13 / 55 (23.6%)	47 / 361 (13.0%)	2.07	1.03 – 4.14
Proteus mirabilis	3 / 16 (18.8%)	57 / 400 (14.2%)	1.39	0.38 – 5.03
Stenotrophomonas maltophilia	5 / 24 (20.8%)	55 / 392 (14.0%)	1.61	0.58 – 4.50
Pseudomonas aeruginosa	4 / 19 (21.1%)	56 / 397 (14.1%)	1.62	0.52 – 5.07
Candida spp.	35 / 235 (14.9%)	25 / 181 (13.8%)	1.09	0.63 – 1.09
Candida albicans	21 / 155 (13.5%)	39 / 261 (14.9%)	0.89	0.50 – 1.58
Pichia kudriavzevii	6 / 15 (40.0%)	54 / 401 (13.5%)	0.98	1.47 – 12.5
Colonization with pathogen: resistant (left column) vs. sensitive/absent (right column) for:				
Amphotericin-B	0 / 1 (0%)	60 / 415 (14.5%)	NA	NA
Fluconazole	13 / 96 (13.5%)	47 / 320 (14.7%)	0.91	0.47 – 1.76
Ciprofloxacin	10 / 58 (17.2%)	47 / 338 (13.9%)	1.29	0.61 – 2.73
TMP/SMX	12 / 81 (14.8%)	45 / 315 (14.3%)	1.04	0.52 – 2.08
Colistin	7 / 41 (17.1%)	50 / 355 (14.1%)	1.26	0.53 – 2.99
Tobramycin	8 / 37 (21.6%)	49 / 359 (13.6%)	1.75	0.75 – 4.04
Penicillin	3 / 15 (20.0%)	54 / 381 (14.2%)	1.51	0.41 – 5.54
**Multivariable model**				
Intensive chemotherapy vs. HSCT			3.81	2.10 – 6.90
Pichia kudravzevii colonization			5.40	1.75 – 16.7
MASCC (per point decrease)			1.16	1.05 – 1.28
Quinolone prophylaxis			0.42	0.20 – 0.92

Abbreviations: AML acute myeloid leukaemia; ECOG Eastern Cooperative Oncology Group; HSCT hematopoietic stem cell transplantation; MASCC Multinational Association for Supportive Care in Cancer; MBI-LCBI mucosal barrier injury-associated laboratory confirmed bloodstream infection; MDS myelodysplastic syndrome; MM multiple myeloma; TMP/SMX trimethoprim-sulfamethoxazole

◊ 2 missing cases

∫ 4 missing cases

Θ 248 valid cases

* oral solution

¶ triazoles consisted of voriconazole, posaconazole or itraconazole

Supplemental Table 5 ─ Univariable and multivariable regression model of candidaemia

Factor	MBI-LCBI		OR	95% CI
**Univariate model**	**Group with factor (n/N.%)**	**Group without factor (n/N.%)**		
Age (years, [mean, 95% CI])	60.9 (55.8 – 65.9)	56.5 (55.3 – 57.7)	1.04	0.97 – 1.12
Male vs. female	6 / 258 (2.3%)	3 / 158 (1.9%)	1.23	0.30 – 4.99
Haematological diagnosis				
AML	5 / 126 (4.0%)	4 / 290 (1.4%)	2.96	0.78 – 11.2
MM	1 / 149 (0.7%)	8 / 267 (3.0%)	0.22	0.03 – 1.77
Lymphoma	0 / 84 (0%)	9 / 332 (2.7%)	NE[§]	NE[§]
MDS	2 / 37 (5.4%)	7 / 379 (1.8%)	3.04	0.61 – 15.2
Other	1 / 20 (5.0%)	8 / 396 (2.0%)	2.55	0.30 – 21.5
Hospital				
Hospital 2 vs. 1	0 / 79 (0%)	6 / 181 (3.3%)	NE[§]	NE[§]
Hospital 3 vs. 1	1 / 43 (2.3%)		0.69	0.08 – 5.92
Hospital 4 vs. 1	2 / 35 (5.7%)		1.77	0.34 – 9.14
Hospital 5 vs. 1	0 / 18 (0%)		NE[§]	NE[§]
Hospital 6 vs. 1	0 / 60 (0%)		NE[§]	NE[§]
Intensive chemotherapy vs. HSCT	7 / 144 (4.9%)	2 / 272 (0.7%)	6.90	1.41 – 33.7
Autologous vs. allogeneic HSCT	1 / 216 (0.5%)	1 / 56 (1.8%)	0.26	0.02 – 4.16
Thrombocyte count ($x10^9$/mL)	15.2 (8.9 – 25.9)	17.2 (15.6 – 19.0)	0.88	0.46 – 1.70
ECOG score[¥]				
1 vs. 0	2 / 72 (2.8%)	2 / 28 (7.1%)	0.37	0.05 – 2.78
2 vs. 0	2 / 134 (1.5%)		0.20	0.03 – 1.46
3 vs. 0	3 / 171 (1.8%)		0.23	0.04 – 1.46
4 vs. 0	0 / 9 (0%)		NE[§]	NE[§]
MASCC (per point decrease [mean. 95% CI])	19.3 (16.5 – 22.2)	19.7 (19.5 – 20.0)	0.95	0.75 – 1.20
MASCC <21 vs. ≥21 points	7 / 322 (2.2%)	2 / 94 (2.1%)	1.02	0.21 – 5.01
Burden of illness				
Moderate vs. mild/no symptoms	2 / 116 (1.7%)	7/290 (2.4%)	0.71	0.15 – 3.47
Severe vs. mild/no symptoms	0 / 10 (0%)		NE[§]	NE[§]
Hypotension	0 / 8 (0%)	9 / 408 (2.2%)	NE[§]	NE[§]
Active COPD	2 / 8 (25%)	7 / 408 (1.7%)	19.1	3.27 - 111.7
Previous fungal infection	0 / 24 (0%)	9 / 392 (2.3%)	NE[§]	NE[§]
Dehydration	4 / 196 (2.0%)	5 / 220 (2.3%)	0.90	0.24 – 3.38
Inpatient vs. outpatient status	8 / 3991 (2.0%)	1 / 25 (4.0%)	0.52	0.06 – 4.17
Age ≥ 60 years	6 / 203 (3.0%)	3 / 213 (1.4%)	2.13	0.53 – 6.64
Fever after day 3	4 / 131 (3.1%)	2 / 110 (1.8%)	1.70	0.31 – 9.47
Oral Mucositis[◊]				
Grade 1-2 vs. 0	1 / 138 (0.7%)	6 / 228 (2.6%)	0.27	0.03 – 2.27
Grade 3-4 vs. 0	2 / 48 (4.2%)		1.61	0.32 – 8.22
Intestinal mucositis[∫]				
Grade 1-2 vs. 0	5 / 243 (2.1%)	2 / 133 (1.5%)	1.38	0.26 – 7.19
Grade 3-4 vs. 0	1 / 27 (3.7%)		2.52	0.22 – 28.8
Citrulline [ϴ] (per □mol/L decrease [mean. 95% CI])	4.01 (2.67 – 5.36)	9.42 (8.70 – 10.12)	1.62	1.12 – 2.33
Citrulline < 10 □mol/L[ϴ]	8 / 156 (5.1%)	0 / 92 (0%)	NE[§]	NE[§]
Prophylaxis				
Amphotericin-B[*]	1 / 84 (1.2%)	8 / 332 (2.4%)	0.49	0.06 – 3.96
Fluconazole	6 / 266 (2.3%)	3 / 150 (2.0%)	1.13	0.28 – 4.59
Triazole [¶]	0 / 79 (0%)	9 / 337 (2.7%)	NE[§]	NE[§]
Colonization cultures				
Escherichia coli	3 / 217 (1.4%)	6 / 199 (3.0%)	0.45	0.11 – 1.83
Klebsiella pneumoniae	1 / 55 (1.8%)	8 / 361 (2.2%)	0.82	0.10 – 6.66
Proteus mirabilis	0 / 16 (0%)	9 / 400 (2.3%)	NE[§]	NE[§]
Stenotrophomonas maltophilia	0 / 24 (0%)	9 / 392 (2.3%)	NE[§]	NE[§]
Pseudomonas aeruginosa	0 / 19 (0%)	9 / 397 (2.3%)	NE[§]	NE[§]
Candida spp.	9 / 235 (3.8%)	0 / 181 (0%)	NE[§]	NE[§]
Candida albicans	5 / 155 (3.2%)	4 / 261 (1.5%)	2.14	0.57 – 8.10
Candida tropicalis	3 / 13 (23.1%)	6 / 403 (1.5%)	19.9	4.34 – 90.9
Pichia kudriavzevii	3 / 15 (20.0%)	6 / 401 (1.5%)	16.5	3.67 – 73.8
Colonization with pathogen: resistant (left column) vs. sensitive/absent (right column) for:				
Amphotericin-B	0 / 1 (0%)	9 / 415 (2.2%)	NE[§]	NE[§]
Fluconazole	5 / 96 (5.2%)	4 / 320 (1.3%)	4.34	1.14 – 16.5

**Multivariable model**		
Active COPD	15.4	1.61 – 147
Fluconazole-resistant Candida colonization	5.85	1.27 – 27.0
Citrulline (per □mol/L decrease)	1.64	1.11 – 2.41

Abbreviations: AML acute myeloid leukaemia; COPD chronic obstructive pulmonary disease; ECOG Eastern Cooperative Oncology Group; HSCT hematopoietic stem cell transplantation; MASCC Multinational Association for Supportive Care in Cancer; MBI-LCBI mucosal barrier injury-associated laboratory confirmed bloodstream infection; MDS myelodysplastic syndrome; MM multiple myeloma.
◊ 2 missing cases
∫ 4 missing cases
Θ 248 valid cases
* oral solution
¶ triazoles consisted of voriconazole, posaconazole or itraconazole
§ not estimable

Supplemental Table 6 ─ Multivariable regression model of bacterial MBI-LCBI pathogens

	**OR (95% confidence interval)**
Chemotherapy vs. SCT	3.13 (1.70 to 5.76)
Pichia kudriavzevii colonization	2.02 (0.53 to 7.71)
MASCC-score (per point decrease)	1.17 (1.06 to 1.30)
Quinolone prophylaxis use	0.48 (0.21 to 1.09)

MBI-LCBI – mucosal barrier injury-associated laboratory confirmed bloodstream infection; SCT – stem cell transplantation; MASCC-score, Multinational Association for Supportive Care in Cancer; OR – odds ratio;

Supplemental Table 7 ─ Multivariable regression model of candidaemia after exclusion of *Pichia kudriavzevii* BSIs

	**OR (95% confidence interval)**
Chemotherapy vs. SCT	3.50 (1.93 to 6.35)
Pichia kudriavzevii colonization	1.86 (0.49 to 7.12)
MASCC-score (per point decrease)	1.18 (1.07 to 1.31)
Quinolone prophylaxis use	0.41 (0.19 to 0.88)

BSI – bloodstream infection; MBI-LCBI – mucosal barrier injury-associated laboratory confirmed bloodstream infection; SCT – stem cell transplantation; MASCC-score, Multinational Association for Supportive Care in Cancer; OR – odds ratio

Supplemental Table 8 – Sensitivity analysis of the incidence of MBI-LCBI: comparison of binomial vs. Poisson GEE

		Period from start of chemotherapy				
		day 1-7	day 8-14	day 15-21	day 22-28	day >28
IRR crude (95% CI)	Poisson	1 (ref.)	6.05 (2.18 to 16.8)	11.2 (3.93 to 32.0)	2.53 (0.57 to 11.2)	2.39 (0.56 to 10.1)
	Binomial	1 (ref.)	6.36 (2.26 to 17.9)	11.7 (4.01 to 33.9)	2.56 (0.56 to 11.6)	2.58 (0.56 to 11.8)
IRR adjusted (95% CI)	Poisson	1 (ref.)	6.05 (2.20 to 16.7)	10.5 (3.66 to 30.1)	2.05 (0.46 to 9.23)	1.83 (0.41 to 8.17)
	Binomial	1 (ref.)	6.56 (2.31 to 18.6)	11.3 (3.82 to 33.6)	2.03 (0.44 to 9.47)	1.96 (0.40 to 9.66)

GEE – generalised estimating equIRR – incidence rate ratio; MBI-LCBI mucosal barrier injury-associated laboratory confirmed bloodstream infection
